# Nomogram for predicting post-percutaneous coronary intervention restenosis in patients with coronary artery disease and diabetes: a retrospective study

**DOI:** 10.7717/peerj.21697

**Published:** 2026-10-13

**Authors:** Ping Wang, Hongyu Peng, Qian Fan, Yuan Shan, Ning Yin

**Affiliations:** Center for Coronary Artery Disease (CCAD), Beijing Anzhen Hospital, Capital Medical University, Beijing Institute of Heart, Lung and Blood Vessel Disease, Beijing, China

**Keywords:** Coronary artery disease, Diabetes mellitus, Restenosis, Percutaneous coronary intervention, Nomogram prediction model

## Abstract

**Background:**

Coronary artery disease (CAD) combined with diabetes mellitus (DM) significantly increases the risk of vascular restenosis following percutaneous coronary intervention (PCI). Despite advancements in stent technology, restenosis remains a concern, especially in diabetic patients. This study aims to predict restenosis risk using serum metabolites and risk factors.

**Methods:**

A retrospective analysis was conducted on 272 CAD and type 2 diabetes mellitus (T2DM) patients who underwent PCI between January 2020 and June 2023. Based on one-year angiographic follow-up, patients were divided into restenosis (*n* = 121) and non-restenosis groups (*n* = 151). Serum metabolites and biomarkers, including glycated hemoglobin (HbA1c), insulin-like growth factor I (IGF-I), low-density lipoprotein cholesterol (LDL-C), tumor necrosis factor-α (TNF-α), and high-sensitivity C-reactive protein (hsCRP), were evaluated, together with procedure-related characteristics (including stent type and post-procedural percentage diameter stenosis) and medication use (including calcium antagonists). Logistic regression and correlation analysis identified significant predictors, and a nomogram prediction model was developed and internally validated using bootstrap resampling.

**Results:**

Lower HbA1c levels were associated with restenosis (rho = −0.142, * P* = 0.008; OR = 0.767, 95% CI [0.625–0.941]). Higher levels of IGF-I (rho = 0.328, *P* < 0.001; OR = 1.053, 95% CI [1.032–1.075]), LDL-C (*r* = 0.135, *P* = 0.025; OR = 1.682, 95% CI [1.107–2.555]), TNF-α (*r* = 0.172, *P* = 0.004; OR = 1.434, 95% CI [1.119–1.838]), and hsCRP (*r* = 0.179, *P* = 0.003; OR = 1.786, 95% CI [1.185–2.693]) were also significantly associated. Non-drug-eluting stents (DES) and higher percentage diameter stenosis (%DS) increased restenosis risk. Calcium antagonists use was negatively correlated with restenosis (*r* =  − 0.185, *P* = 0.002; OR = 0.449, 95% CI [0.228–0.884]). The nomogram model demonstrated good discrimination for restenosis (apparent AUC = 0.833; bootstrap-corrected AUC = 0.808).

**Conclusion:**

This study identified key predictive factors for post-PCI restenosis in CAD patients with DM through case-control analysis. The nomogram model showed good discrimination on internal bootstrap validation. Pending external validation, it may provide a useful exploratory tool to support risk stratification and inform management aimed at reducing the restenosis burden in high-risk cardiovascular populations.

## Introduction

Coronary artery disease (CAD) remains a leading cause of morbidity and mortality worldwide, with approximately 7.3 million deaths annually attributed to CAD, accounting for 13% of global total deaths. The prevalence of CAD is exacerbated by comorbid metabolic conditions such as diabetes mellitus (DM). Data indicate that individuals with diabetes have a 2 to 4 times higher risk of developing coronary heart disease compared to those without diabetes (Zhang et al., 2022). Percutaneous coronary intervention (PCI) has emerged as an integral therapeutic strategy for the revascularization in CAD, offering symptom relief and prognosis improvement (Yerasi et al., 2020). However, postprocedural complications, specifically vascular restenosis, present substantial clinical challenges, particularly among patients with DM who exhibit an inherently elevated risk (Xu et al., 2022). The pathophysiology of restenosis involves neointimal hyperplasia, influenced by endothelial damage and inflammatory and proliferative cellular responses, often exacerbated in diabetic states due to persistent endothelial dysfunction and heightened inflammatory milieu (Xu et al., 2023). Therefore, identifying relevant biomarkers is crucial for the treatment of patients experiencing restenosis following PCI.

DM raises the risk of restenosis after percutaneous coronary intervention. DM significantly affects atherosclerotic plaque composition through mechanisms involving advanced glycation end products (AGEs), oxidative stress, and altered lipid profiles (Xenogiannis et al., 2021). These alterations facilitate both the progression of atherosclerosis and the heightened propensity for restenosis post-PCI (Wybraniec et al., 2021). Thus, a promising line of inquiry is to use serum metabolites, together with inflammatory and procedural variables, to identify patients at high risk of vascular restenosis following PCI in patients with CAD and DM, thereby supporting earlier and more individualized risk stratification rather than treatment *per se*.

The multifactorial nature of restenosis was well-documented, with contributory roles played by metabolic dysregulation, inflammation, and the mechanical factors relating to stent implantation (Wang et al., 2022). The prevalence of type 2 DM, characterized by insulin resistance and hyperglycemia, necessitates a nuanced understanding of these factors to tailor interventions effectively (Vargas-Alarcón et al., 2022). Despite the evolution of stent technology, including the development of drug-eluting stents (DES), restenosis remains a persistent concern (Sun et al., 2020). While DES reduces restenotic rates by inhibiting neointimal growth *via* localized drug release, variability in patient responses signifies the complexity of biological interactions influencing outcomes (Spadaccio et al., 2020). This heterogeneity, particularly pronounced in diabetic populations, underscores the need for a predictive model that assimilates diverse risk factors to enhance prognostication and intervention strategies (Sharifi et al., 2023).

Serum metabolites, cytokine concentrations, and medications all influence the development of CAD and the occurrence of in-stent restenosis following PCI to varying degrees. Understanding these relationships can enable more precise risk assessment for restenosis after PCI. Abnormal levels of serum metabolites such as high-density lipoprotein cholesterol (HDL-C), low-density lipoprotein cholesterol (LDL-C), and triglycerides (TG) not only increase the risk of coronary artery disease (CAD) but can also promote neointimal hyperplasia after percutaneous coronary intervention (PCI), leading to restenosis. A hyperglycemic state accelerates the progression of atherosclerosis and increases the incidence of post-PCI complications. Serum concentrations of cytokines, as key regulators of the immune system, play a crucial role in inflammatory responses and directly impact the development of CAD and the healing process after PCI. Pro-inflammatory cytokines can stimulate vascular smooth muscle cell proliferation and migration, increasing fibrous tissue formation and promoting post-PCI restenosis. Anti-inflammatory cytokines help mitigate inflammatory responses and reduce the occurrence of restenosis. Medications play a central role in managing and preventing CAD and its post-PCI complications. Statins lower cholesterol levels, stabilize plaques, and have anti-inflammatory effects; antiplatelet drugs, such as aspirin and clopidogrel, are used to prevent thrombosis; novel antidiabetic medications not only control blood glucose but also exhibit cardiovascular protective effects, reducing the risk of major adverse cardiovascular events like myocardial infarction and stroke. Given the current state of research into diabetes, coronary heart disease and restenosis following percutaneous coronary intervention, it is crucial to identify reliable risk factors associated with these conditions. We therefore hypothesized that a defined panel of serum metabolites, inflammatory biomarkers, procedural characteristics, and medication use is associated with post-PCI restenosis in patients with CAD and T2DM, and that these factors can be integrated into a multivariable nomogram for risk prediction. Accordingly, the primary aim of this study was to identify factors independently associated with restenosis using a retrospective case-control approach and to construct a nomogram for predicting post-PCI restenosis risk in this population. Because the design is observational, the identified associations are intended to support risk stratification and to generate hypotheses for future interventional research, rather than to establish causal treatment effects.

## Materials and Methods

### Study design and population

A retrospective analysis was conducted using the medical records of 272 patients with CAD and diabetes, who were hospitalized at our institution between January 2020 and June 2023. Ethical approval was obtained from Beijing Anzhen Hospital (Ethics Approval Number: 2026041X); details of the informed-consent waiver are provided in the following paragraph. Patient data were retrieved from the hospital’s medical record system. Inclusion criteria: (1) Participants were over 18 years of age (2) Participants had type 2 DM (T2DM) confirmed to have two or three-vessel CAD. The diagnoses of T2DM and CAD were established using the standards set by the American Diabetes Association (ElSayed et al., 2023) and the American Heart Association (Arnold et al., 2020), HbA1c ≥ 6.5%, coronary artery stenosis ≥ 50%. (3) Eligible patients must have undergone PCI surgery (4) with comprehensive coronary angiography performed both pre- and post-PCI yielding clear and complete imaging data. Additionally; (5) patients needed to have comprehensive medical records and data preservation. Exclusion criteria: (1) Participants had used any supplements, such as magnesium and/or zinc, within three months prior to the study, or if they had used antioxidants, anti-inflammatory supplements, or *ω*-3 fatty acids, (2) the presence of immune system diseases, infections, tumors, hematological disorders, severe liver or kidney dysfunction, myocarditis, pericarditis, cardiomyopathy, congenital heart disease, valvular heart disease, or other structural heart diseases. (3) Thyroid disease, recent infections, (4) the use of antibiotics during the follow-up period, (5) loss to follow-up or absence of follow-up within one year after PCI, (6) cognitive impairment. The patients were categorized into two groups based on the occurrence of vascular restenosis following PCI. Vascular restenosis was defined as a diameter stenosis of 50% or greater on angiography at the one-year follow-up. This includes both the diameter within the stent and the diameter extending five mm proximally and distally from the stent. The no-restenosis group included 151 patients, while the restenosis group comprised 121 patients.

This study was approved by our institution’s Ethics Committee and Institutional Review Board. Due to the retrospective nature of the research, obtaining informed consent is not required. The research relies on de-identified patient data to protect privacy and eliminate any potential risks or effects on patient care. This exemption aligns with regulatory standards and ethical principles relevant to retrospective research. The study strictly adhered to the ethical guidelines set forth by Institutional Review Boards and the updated provisions of the 1964 Declaration of Helsinki, as revised in 2013.

### PCI and postoperative medication

All PCIs were executed by a team of 2 to 3 experienced interventional cardiologists. Prior to emergency procedures, patients received an oral dose of 300 mg of enteric-coated aspirin (product code: HJ20160685, Bayer HealthCare Manufacturing S.r.l., Italy) and 300 mg of clopidogrel (product code: H20123116, Lepu Medical Co., Ltd., China). In contrast, for elective procedures, patients were administered 100 mg/day of aspirin (same product) and 75 mg/day of clopidogrel (same product) for three consecutive days leading up to the surgery. During the PCI, patients were positioned supine, and access was obtained *via* the radial or femoral artery using the Seldinger technique. Local infiltration anesthesia with lidocaine (product code: H20043676, Sinopharm Group Rongsheng Pharmaceutical Co., Ltd., China) was applied subcutaneously at the puncture site. For anticoagulation, a dose of 8,000 to 10,000 units of heparin (product code: H32020612, Jiangsu Wanbang Shenghua Medicine Co., Ltd., China) was administered through the arterial sheath, with the exact dosage adjusted for body weight. Postoperatively, patients were given 4,000 IU of low molecular weight heparin (product code: H20040409, Qilu Pharmaceutical Co., Ltd., China) subcutaneously twice daily for 3 to 5 days. They continued oral administration of 100 mg of aspirin and 75 mg of clopidogrel daily for a minimum of 12 months. The procedure was deemed successful if the residual stenosis was less than 10% according to visual estimation in at least two orthogonal projections, the distal vessel of the stent achieved TIMI 3 flow, and there were no major procedure-related complications, such as myocardial infarction, sudden death, or emergency coronary artery bypass grafting. One year post-PCI, all patients returned for routine follow-up examinations. The following stent details were recorded: number of stents used, number of overlapping stents, total length of stents, average diameter of stents, and type of stents. At one-year follow-up, the medication usage of patients was recorded, including β-blockers, calcium antagonists, ACE inhibitors or ARBs, long-acting nitrates, diuretics, digoxin, warfarin, lipid-lowering drugs, aspirin, and others.

### Measurement of serum metabolites

Fasting blood samples for serum metabolites were collected prior to PCI. Serum insulin levels were quantified using Enzyme-Linked Immunosorbent Assay (ELISA) provided by DAKO, Hamburg, Germany, and serum proinsulin concentrations were determined using a two-site ELISA from Mercodia, Uppsala, Sweden. Leptin levels were analyzed *via* a double-antibody radioimmunoassay (RIA) from Linco Research, St. Louis, MO, USA, with a coefficient of variation (CV) of 4.7% and a concentration range of 2–15 ng/mL. Serum total Insulin-like Growth Factor I (IGF-I) was measured in micrograms per liter (µg/L) using RIA, following acid-ethanol extraction and cold precipitation to separate IGFs from IGF binding proteins (IGFBPs), using des (1–3) IGF-I as the radioligand to minimize residual IGFBPs interference. HbA1c levels were determined through high-performance liquid chromatography (HPLC) using capillary blood collected on filter paper, with the upper normal limit set at 5.3%. This analysis was facilitated by Boehringer Mannheim Scandinavian AB, based in Bromma, Sweden. HDL-C and LDL-C concentrations were measured with the Beckman Coulter AU680 automated biochemical analyzer from Beckman (Brea, CA, USA). The Homeostatic Model Assessment of Insulin Resistance (HOMA-IR) was calculated using the formula: HOMA-I*R* = (Fasting Glucose × Fasting Insulin)/405.

### Measurement of biomarkers

Fasting blood samples for various biomarkers were collected prior to PCI. Serum concentrations of cytokines, including INF-γ, IL-1β, IL-6, and TNF-α, were quantified using a multiplex bead-based suspension enzyme-linked immunosorbent assay (ELISA) provided by Biosource, conducted on the Luminex 100 system (Luminex Corp., Austin, TX, USA). The assay had a detection limit of one pg/mL, and the inter-assay coefficient of variation (CV) ranged from 5% to 10%. N-terminal pro-brain natriuretic peptide (NT-proBNP) was measured using an electrochemiluminescence immunoassay (ECLIA) within a detection range of 5 to 35,000 ng/L, utilizing the Modular EE system from Roche. High-sensitivity C-reactive protein (hsCRP) levels were determined by an immunoturbidimetric rate method (Turbidimeri, NIPIA rate), with a detection range of 0.2 to 380 mg/L.

### Angiography

Coronary angiography was conducted before and after successful surgery and at the one-year follow-up. Quantitative coronary angiography (QCA) was performed using the CAAS 5.9 system (Pie Medical Imaging BV, Maastricht, Netherlands). Multiple views of coronary angiograms were recorded following the intracoronary administration of nitroglycerin. Key measurements included reference diameter (RD), minimal lumen diameter (MLD), percentage diameter stenosis (%DS), and lesion length. Acute gain-1 was defined as the difference between the MLD immediately after the initial ballooning and the MLD at baseline. Acute gain-2 was calculated as the difference between the MLD immediately after PCI balloon angioplasty and the MLD post-initial ballooning. The dilation ratio during the initial ballooning was determined by dividing the MLD after initial ballooning by the RD at baseline.

### Statistical analysis

Data were analyzed using SPSS statistical software version 29.0 (SPSS Inc., Chicago, IL, USA). Categorical data were presented as numbers and percentages [n (%)]. Continuous variables were initially assessed for normal distribution with the Shapiro–Wilk test. If normally distributed, continuous data were expressed as mean ± standard deviation (SD). A *P*-value of less than 0.05 was considered indicative of statistical significance. To examine the relationship between serum metabolites, risk factors, and vascular restenosis following PCI in patients with CAD and DM, correlation analysis, logistic regression, and factor analysis were employed. Additionally, a Receiver Operating Characteristic (ROC) curve was plotted, the area under the curve (AUC) was calculated, and a nomogram prediction model was constructed using the predictors that remained statistically significant in the multivariable logistic regression model. To evaluate the internal stability of the model and to correct for optimism arising from developing and testing the model on the same dataset, internal validation was conducted using bootstrap resampling with 1,000 replications, and an optimism-corrected AUC was derived. The ROC curve was plotted using R version 4.0.0. AUC > 0.8 is considered a threshold for good predictive performance; a 95% confidence interval (CI) that excludes 1 indicates statistical significance, while a CI that includes 1 suggests the statistic is not significant. An odds ratio (OR) >1 indicates a positive correlation between serum metabolites, influence factor, and restenosis, while an OR <1 indicates a negative correlation between serum metabolites, influence factor, and restenosis.

## Results

### Demographic and basic data

A total of 272 patients including 151 patients with no-restenosis group and 121 patients with restenosis group were included (Table 1). There was no statistically significant difference in the sex distribution between the two groups (*P* = 0.994), with both groups having a similar proportion of males and females. The mean age was slightly higher in the restenosis group (63.35 ±  6.27 years) compared to the no-restenosis group (62.27 ± 8.36 years), though this difference was not statistically significant (*P* = 0.225). Body mass index (BMI) values were comparable across the groups (*P* = 0.512). Additionally, other factors such as smoking status (*P* = 0.136), left ventricular ejection fraction (*P* = 0.754), and lesion characteristics, including calcifications (*P* = 0.217) and bifurcation presence (*P* = 0.249), showed no significant differences. Characteristics related to diabetes, including the duration of diabetes (*P* = 0.847) and micro-vascular complications (*P* = 0.104), were similar between the groups. Treatment with glucose-lowering medications, like acarbose (*P* = 0.486) and metformin (*P* = 0.321), showed no significant differences. Angiographic findings before the procedure, such as lesion length (*P* = 0.254) and percentage diameter stenosis (%DS) (*P* = 0.714), were also similar across both groups. These results indicate that there were no significant differences in baseline demographic and clinical characteristics between patients who experienced restenosis after PCI and those who did not. This allows for subsequent case-control analyses between the groups.

**Table 1:** Baseline demographic data.

**Parameters**	**No-restenosis (*n* = 151)**	**Restenosis (*n* = 121)**	**t/*χ*** ^**2**^	***P***
Male/Female	111 (73.51%)/40 (26.49%)	89 (73.55%)/32 (26.45%)	0.000	0.994
Age (years)	62.27 ± 8.36	63.35 ± 6.27	1.217	0.225
BMI (kg/m)	28.34 ± 5.23	27.96 ± 4.38	0.656	0.512
Smoking	93 (61.59%)	85 (70.25%)	2.227	0.136
LVEF (%)	58.36 ± 13.08	57.85 ± 13.51	0.314	0.754
Calcifications (%)	49 (32.45%)	48 (39.67%)	1.526	0.217
Blunt stump (%)	64 (42.38%)	65 (53.72%)	3.461	0.063
Tortuosity (%)	12 (7.95%)	13 (10.74%)	0.63	0.428
Bifurcation (%)	22 (14.57%)	12 (9.92%)	1.329	0.249
Retrograde approach (%)	43 (28.48%)	46 (38.02%)	2.777	0.096
Diabetes				
Duration (years)	6.34 ± 2.04	6.29 ± 2.16	0.193	0.847
Micro-vascular complications	11 (7.28%)	16 (13.22%)	2.649	0.104
Glucose lowering treatment				
Acarbose	6 (3.97%)	7 (5.79%)	0.484	0.486
Sulphonylurea	79 (52.32%)	72 (59.5%)	1.405	0.236
Insulin	54 (35.76%)	52 (42.98%)	1.47	0.225
Metformin	60 (39.74%)	41 (33.88%)	0.985	0.321
Angiographic findings				
Before procedure Lesion length (mm)	19.26 ± 5.13	20.25 ± 8.26	1.145	0.254
Before procedure %DS (%)	82.16 ± 11.27	82.65 ± 10.39	0.367	0.714
Before procedure MLD (mm)	0.51 ± 0.12	0.5 ± 0.15	0.579	0.563
History of hypertension	92 (60.93%)	78 (64.46%)	0.358	0.549
History of hyperlipidemia	53 (35.1%)	49 (40.5%)	0.835	0.361
Family history of CHD	12 (7.95%)	12 (9.92%)	0.324	0.569

**Notes.**

%DS Percentage Diameter Stenosis MLD Minimum Lumen Diameter CHD Coronary Heart Disease

### Serum metabolites

The restenosis group exhibited a lower mean HbA1c level (6.29% ± 1.09%) compared to the no-restenosis group (6.69% ±  1.26%) (*P* = 0.007) (Table 2). Furthermore, the mean level of IGF-I was significantly higher in the restenosis group (108.33 ± 12.84 µg/L) compared to the no-restenosis group (98.39 ± 13.71 µg/L), with a *P* value of less than 0.001. Additionally, LDL cholesterol levels were elevated in the restenosis group (2.71 ± 0.51 mmol/L) when compared to the no-restenosis group (2.52 ± 0.63 mmol/L), and this difference was statistically significant (*P* = 0.008). Other serum metabolites, such as glucose (*P* = 0.371), insulin (*P* = 0.091), proinsulin (*P* = 0.24), HOMA-IR (*P* = 0.238), leptin (*P* = 0.355), and HDL cholesterol (*P* = 0.783), did not demonstrate statistically significant differences between the two groups. These findings highlight specific metabolic differences associated with restenosis in patients undergoing percutaneous coronary intervention (PCI), including high levels of IGF-I and LDL cholesterol, as well as low levels of HbA1c.

**Table 2:** Comparison of serum metabolites between the two groups.

**Parameters**	**No-restenosis (*n* = 151)**	**Restenosis (*n* = 121)**	**t/*χ*** ^**2**^	***P***
Glucose (mmol/L)	7.25 ± 1.81	7.42 ± 1.36	0.896	0.371
HbA1c (%)	6.69 ± 1.26	6.29 ± 1.09	2.706	0.007
IGF-I (µg/L)	98.39 ± 13.71	108.33 ± 12.84	6.111	<0.001
Insulin (µU/mL)	15.62 ± 2.13	16.29 ± 3.91	1.701	0.091
Proinsulin (pmol/L)	29.32 ± 2.67	28.95 ± 2.42	1.179	0.24
HOMA-IR (mmol/L* µU/mL)	5.54 ± 0.97	5.68 ± 0.89	1.183	0.238
Leptin (ng/mL)	14.35 ± 3.16	14.66 ± 2.48	0.926	0.355
LDL cholesterol (mmol/L)	2.52 ± 0.63	2.71 ± 0.51	2.679	0.008
HDL cholesterol (mmol/L)	1.06 ± 0.35	1.08 ± 0.26	0.276	0.783

**Notes.**

HbA1c Glycated Hemoglobin A1c IGF-I Insulin-like Growth Factor I HOMA-IR Homeostatic Model Assessment for Insulin Resistance LDL Low-Density Lipoprotein HDL High-Density Lipoprotein

### Biomarkers

The levels of tumor necrosis factor α (TNF-α) and high-sensitivity C-reactive protein (hsCRP) were higher in the restenosis group, at 8.21 ± 1.11 pg/mL and 3.56 ± 0.58 mg/L, respectively, compared to the no-restenosis group, which had levels of 7.82 ± 0.93 pg/mL and 3.33 ± 0.62 mg/L. This difference was statistically significant, with *P* values of 0.002 for TNF-α and *P* = 0.003 for hsCRP (Table 3). Other biomarkers, including interferon-gamma (IFN-γ) (*P* = 0.057), interleukin-1 beta (IL-1b) (*P* = 0.414), interleukin-6 (IL-6) (*P* = 0.087), and the levels of N-terminal pro-B-type natriuretic peptide (NT proBNP) (*P* = 0.315) showed no significant differences between the groups. These findings highlight the link between elevated TNF-α and hsCRP levels and the occurrence of restenosis after PCI in patients with CAD and DM. High levels of TNF-α and hsCRP can serve as predictors of restenosis after PCI.

**Table 3:** Comparison of biomarkers between the two groups.

**Parameters**	**No-restenosis (*n* = 151)**	**Restenosis (*n* = 121)**	**t/*χ*** ^**2**^	***P***
IFN-γ (pg/mL)	14.84 ± 1.48	15.26 ± 2.03	1.916	0.057
IL-1b (pg/mL)	1.03 ± 0.31	1.06 ± 0.36	0.818	0.414
IL-6 (pg/mL)	8.96 ± 1.03	9.21 ± 1.24	1.719	0.087
TNF-α (pg/mL)	7.82 ± 0.93	8.21 ± 1.11	3.106	0.002
hsCRP (mg/L)	3.33 ± 0.62	3.56 ± 0.58	3.015	0.003
NT proBNP (ng/L)	315.27 ± 50.26	322.15 ± 60.23	1.007	0.315

**Notes.**

IFN-γ Interferon-gamma IL-1b Interleukin-1 beta IL-6 Interleukin-6 TNF-α Tumor Necrosis Factor-alpha hsCRP High-Sensitivity C-Reactive Protein NT proBNP N-terminal pro-B-type Natriuretic Peptide

### Stent usage characteristics

The total stenting length was notably greater in the restenosis group (83.25 ± 23.19 mm) compared to the no-restenosis group (73.96 ± 26.47 mm), with statistical significance achieved (*P* = 0.003) (Table 4). Furthermore, the type of stent used significantly differed between the groups (*P* = 0.001), with the no-restenosis group having a higher proportion of DES usage (88.08%) compared to the restenosis group (75.21%), while non-DES stents were more frequently utilized in the restenosis group (24.79%) compared to the no-restenosis group (11.92%). There were no significant differences between the two groups in terms of the number of stents used (*P* = 0.164), the incidence of overlapping stents (*P* = 0.14), and the mean stent diameter (*P* = 0.5). These findings emphasize that the use of non-drug-eluting stents (non-DES) and longer stents may increase the risk of restenosis after PCI.

**Table 4:** Comparison of stent usage between the two groups.

**Parameters**	**No-restenosis (*n* = 151)**	**Restenosis (*n* = 121)**	**t/*χ*** ^**2**^	***P***
Number of stents			1.935	0.164
≤2	45 (29.8%)	27 (22.31%)		
>2	106 (70.2%)	94 (77.69%)		
Overlapping stent (%)	138 (91.39%)	116 (95.87%)	2.179	0.14
Stenting total length (mm)	73.96 ± 26.47	83.25 ± 23.19	3.037	0.003
Stent mean diameter (mm)	2.83 ± 0.46	2.89 ± 0.75	0.676	0.5
Stent type, n (%)			8.464	0.004
DES	133 (88.08%)	91 (75.21%)		
Others	18 (11.92%)	30(24.79%)		

**Notes.**

BMS Bare Metal Stents DES Drug-Eluting Stents

following PCI in patients with CAD and DM.

### Balloon angioplasty parameters and postoperative vascular response

After the procedure, the percentage diameter stenosis (%DS) was notably higher in the restenosis group (13.27% ± 4.39%) compared to the no-restenosis group (11.69% ± 3.25%), with this difference being statistically significant (*P* = 0.001) (Table 5). Similarly, the minimum lumen diameter (MLD) measured after the procedure was significantly smaller in the restenosis group (2.16 ± 0.58 mm) compared to the no-restenosis group (2.38 ± 0.63 mm), achieving statistical significance (*P* = 0.003). The acute gain-1, representing immediate post-procedural lumen enlargement, was also significantly lower in the restenosis group (1.42 ± 0.56 mm) *versus* the no-restenosis group (1.61 ± 0.39 mm) with a *P* value of 0.002. Furthermore, the expansion rate after initial ballooning was significantly reduced in the restenosis group (75.28% ± 14.09%) as compared to the no-restenosis group (80.16% ± 13.52%), with a *P* value of 0.004. In contrast, no significant differences were observed in initial ballooning parameters such as balloon diameter (*P* = 0.946), balloon length (*P* = 0.415), and inflation pressure (*P* = 0.414), nor in acute gain-2 (*P* = 0.123). These findings indicate that higher procedural %DS (percent diameter stenosis) and lower procedural minimal lumen diameter (MLD), as well as lower Acute gain-1 and expansion rate after initial ballooning, are critical factors in the development of restenosis.

**Table 5:** Comparison of surgical factors between the two groups.

**Parameters**	**No-restenosis (*n* = 151)**	**Restenosis (*n* = 121)**	**t/*χ*** ^**2**^	***P***
Initial ballooning				
Balloon diameter (mm)	2.86 ± 0.38	2.85 ± 0.46	0.068	0.946
Balloon length (mm)	11.71 ± 1.63	11.87 ± 1.67	0.817	0.415
Inflation pressure (atm)	16.62 ± 4.67	17.03 ± 3.65	0.819	0.414
After procedure %DS (%)	11.69 ± 3.25	13.27 ± 4.39	3.293	0.001
After procedure MLD (mm)	2.38 ± 0.63	2.16 ± 0.58	3.021	0.003
Acute gain-1 (mm)	1.61 ± 0.39	1.42 ± 0.56	3.079	0.002
Acute gain-2 (mm)	0.28 ± 0.09	0.29 ± 0.06	1.548	0.123
Expansion rate after initial ballooning (%)	80.16 ± 13.52	75.28 ± 14.09	2.903	0.004

### Postoperative medication usage

The restenosis group had a markedly lower usage of calcium antagonists (20.66%) compared to the no-restenosis group (38.41%), with this difference being statistically significant (*P* = 0.002) (Table 6). Other medications, including beta blockers (*P* = 0.167), ACE inhibitors or angiotensin receptor blockers (ACE or ARB) (*P* = 0.238), long-acting nitrates (*P* = 0.261), diuretics (*P* = 0.735), digitalis (*P* = 0.33), warfarin (*P* = 0.539), lipid-lowering agents (*P* = 0.087), and aspirin (*P* = 0.468), did not show statistically significant differences in usage between the groups. These findings indicate that the low prescription rate of calcium antagonists is associated with restenosis.

**Table 6:** Comparison of medications between the two groups.

**Parameters**	**No-restenosis (*n* = 151)**	**Restenosis (*n* = 121)**	**t/*χ*** ^**2**^	***P***
Beta blockers	123 (81.46%)	106 (87.6%)	1.907	0.167
Calcium antagonists	58 (38.41%)	25 (20.66%)	9.981	0.002
ACE or ARB	63 (41.72%)	42 (34.71%)	1.393	0.238
Long-acting nitrates	95 (62.91%)	84 (69.42%)	1.264	0.261
Diuretics	36 (23.84%)	31 (25.62%)	0.114	0.735
Digitalis	3 (1.99%)	0 (0%)	0.951	0.33
Warfarin	7 (4.64%)	3 (2.48%)	0.378	0.539
Lipid-lowering agent	106 (70.2%)	96 (79.34%)	2.936	0.087
Aspirin	129 (85.43%)	107 (88.43%)	0.526	0.468

**Notes.**

ACE or ARB ACE inhibitor or Angiotensin Receptor Blocker

### Correlation analysis between serum metabolites and risk factors and restenosis following PCI

The correlation analysis identified several factors significantly associated with vascular restenosis following PCI in patients with CAD and DM (Table 7). Positive correlations were observed between restenosis following PCI with IGF-I (rho = 0.328, *P* < 0.001), low-density LDL cholesterol (rho = 0.135, *P* = 0.025), TNF-α (rho = 0.172, *P* = 0.004), high-sensitivity hsCRP (rho = 0.179, *P* = 0.003), stenting total length (rho = 0.158, *P* = 0.009), stent type with DES considered zero (rho = 0.195, *P* = 0.001), and post-procedure percentage diameter stenosis (%DS) (rho = 0.175, *P* = 0.004). Conversely, negative correlations were identified for glycated HbA1c (rho = −0.142, *P* = 0.018), MLD post-procedure (rho = −0.179, *P* = 0.003), acute gain-1 (rho = −0.192, *P* = 0.001), expansion rate after initial ballooning (rho = −0.147, *P* = 0.015), and usage of calcium antagonists (rho = −0.185, *P* = 0.002). These results emphasize the complex relationship between metabolic factors, inflammatory markers, and procedural characteristics in predicting restenosis. The findings indicate that higher levels of IGF-I, LDL cholesterol, TNF-α, hsCRP, stenting total length, stent type (with DES considered as zero), and post-procedure %DS are positively correlated with vascular restenosis after PCI in patients with CAD and DM. Higher levels of these factors suggest a greater risk of restenosis following PCI. Conversely, lower levels of HbA1c, post-procedure MLD, Acute gain-1, expansion rate after initial ballooning, and the use of calcium antagonists are negatively correlated with vascular restenosis after PCI in patients with CAD and DM. Lower levels of these factors suggest a reduced risk of restenosis following PCI. These results provide critical insights for developing a nomogram prediction model.

**Table 7:** Correlation analysis of factors related to vascular restenosis after PCI in patients with CAD and DM.

**Influencing factors**	**rho**	***P***
HbA1c (%)	−0.142	0.018
IGF-I (µg/L)	0.328	<0.001
LDL cholesterol (mmol/L)	0.135	0.025
TNF-α (pg/mL)	0.172	0.004
hsCRP (mg/L)	0.179	0.003
Stenting total length (mm)	0.158	0.009
Stent type, n (%) (Others, DES:0)	0.195	0.001
After procedure %DS (%)	0.175	0.004
After procedure MLD (mm)	−0.179	0.003
Acute gain-1 (mm)	−0.192	0.001
Expansion rate after initial ballooning (%)	−0.147	0.015
Calcium antagonists	−0.185	0.002

### Logistic regression analysis

Univariate logistic regression revealed key factors significantly linked to vascular restenosis after PCI in CAD patients with DM (Table 8). Lower levels of HbA1c (coefficient = −0.265, *P* = 0.012; OR = 0.767, 95% CI [0.625–0.941]), MLD (coefficient = −0.590, *P* = 0.004; OR = 0.554, 95% CI [0.366–0.839]), Acute gain-1 (coefficient = −0.800, *P* = 0.003; OR = 0.449, 95% CI [0.261–0.772]), expansion rate after initial ballooning (coefficient = −0.025, *P* = 0.007; OR = 0.975, 95% CI [0.958–0.993]), and the use of calcium antagonists (coefficient = −0.850, *P* = 0.003; OR = 0.427, 95% CI [0.243–0.750]) were associated with a reduced risk of restenosis. Conversely, higher levels of IGF-I (coefficient = 0.052, *P* < 0.001; OR = 1.053, 95% CI [1.032–1.075]), LDL cholesterol (coefficient = 0.520, *P* = 0.015; OR = 1.682, 95% CI [1.107–2.555]), TNF-α (coefficient = 0.360, *P* = 0.005; OR = 1.434, 95% CI [1.119–1.838]), and hsCRP (coefficient = 0.580, *P* = 0.006; OR = 1.786, 95% CI [1.185–2.693]), post-procedure %DS (coefficient = 0.105, *P* = 0.002; OR = 1.111, 95% CI [1.039–1.188]), total stent length (coefficient = 0.015, *P* = 0.005; OR = 1.014, 95% CI [1.004–1.024]), and the use of certain stent types (coefficient = 0.530, *P* = 0.003; OR = 1.699, 95% CI [1.199–2.408]) were associated with an increased likelihood of restenosis. These findings from univariate analysis informed variable selection for the multivariate model.

**Table 8:** Univariate logistic regression analysis of factors related to vascular restenosis after PCI in patients with CAD and DM.

**Influencing factors**	**Coefficient**	**Std error**	***P***	**OR**	**95%CI**
HbA1c (%)	−0.265	0.112	0.012	0.767	0.625–0.941
IGF-I (µg/L)	0.052	0.010	<0.001	1.053	1.032–1.075
LDL cholesterol (mmol/L)	0.520	0.214	0.015	1.682	1.107–2.555
TNF-α (pg/mL)	0.360	0.127	0.005	1.434	1.119–1.838
hsCRP (mg/L)	0.580	0.210	0.006	1.786	1.185-2.693
Stenting total length (mm)	0.014	0.005	0.005	1.014	1.004–1.024
Stent type, n (%) (Others, DES)	0.530	0.180	0.003	1.699	1.199–2.408
After procedure %DS (%)	0.105	0.034	0.002	1.111	1.039–1.188
After procedure MLD (mm)	−0.590	0.205	0.004	0.554	0.366–0.839
Acute gain-1 (mm)	−0.800	0.205	0.003	0.449	0.261–0.772
Expansion rate after initial ballooning (%)	−0.025	0.009	0.007	0.975	0.958–0.993
Calcium antagonists	−0.850	0.283	0.003	0.427	0.243–0.750

The multivariate logistic regression analysis of factors influencing post-PCI vascular restenosis in patients with CAD and DM identified significant predictors (Table 9). Lower HbA1c levels were inversely associated with restenosis risk (Coefficient = −0.330, *P* = 0.015; OR = 0.719, 95% CI [0.551–0.938]). An increase in IGF-I levels significantly raised the risk of restenosis (Coefficient = 0.058, *P* < 0.001; OR = 1.060, 95% CI [1.034–1.086]). Similarly, TNF-α was a significant positive predictor (Coefficient = 0.400, *P* = 0.010; OR = 1.492, 95% CI [1.098–2.027]). For procedural factors, the type of stent used was significant, with non-DES stents associated with higher restenosis (Coefficient = 0.600, *P* = 0.008; OR = 1.822, 95% CI [1.171–2.835]), while an increased %DS post-procedure also elevated risk (Coefficient = 0.085, *P* = 0.035; OR = 1.089, 95% CI [1.006–1.178]). Conversely, larger post-procedural MLD was protective (Coefficient = −0.750, *P* = 0.003; OR = 0.472, 95% CI [0.285–0.782]), as was greater acute gain-1 (Coefficient = −0.690, *P* = 0.015; OR = 0.502, 95% CI [0.269–0.937]). The expansion rate post-initial ballooning inversely correlated with restenosis (Coefficient = −0.028, *P* = 0.011; OR = 0.972, 95% CI [0.950–0.995]), and the use of calcium antagonists appeared protective (Coefficient = −0.800, *P* = 0.020; OR = 0.449, 95% CI [0.228–0.884]). Other factors, including LDL cholesterol, hsCRP, and total stenting length, did not achieve statistical significance, but the patterns observed suggest complex interactions warranting further investigation. Based on the above results, HbA1c, post-procedure MLD (Minimal Lumen Diameter), Acute gain-1, expansion rate after initial ballooning, and the use of calcium antagonists are protective factors for vascular restenosis after PCI in patients with CAD and DM. As the values of these variables increase, the probability of vascular restenosis decreases. IGF-I, LDL cholesterol, TNF-α, hsCRP, stenting total length, stent type (n %), and post-procedure %DS are risk factors for vascular restenosis after PCI in patients with CAD and DM. As the values of these variables increase, the probability of vascular restenosis also increases. These significant variables inform the nomogram development for predicting restenosis risk.

**Table 9:** Multivariate logistic regression analysis of factors related to vascular restenosis after PCI in patients with CAD and DM.

**Influencing factors**	**Coefficient**	**Std error**	**Wald stat**	***P***	**OR**	**95%CI**
HbA1c (%)	−0.330	0.136	−2.566	0.015	0.719	0.551–0.938
IGF-I (µg/L)	0.058	0.013	4.826	<0.001	1.060	1.034–1.086
LDL cholesterol (mmol/L)	0.170	0.273	0.623	0.533	1.186	0.694–2.025
TNF-α (pg/mL)	0.400	0.155	2.663	0.010	1.492	1.098–2.027
hsCRP (mg/L)	0.315	0.257	1.228	0.220	1.370	0.829–2.267
Stenting total length (mm)	0.009	0.006	1.466	0.143	1.009	0.997–1.021
Stent type, n (%) (Others,DES)	0.600	0.225	2.748	0.008	1.822	1.171–2.835
After procedure %DS (%)	0.085	0.040	2.203	0.035	1.089	1.006–1.178
After procedure MLD (mm)	−0.750	0.255	−3.026	0.003	0.472	0.285–0.782
Acute gain-1 (mm)	−0.690	0.318	−2.248	0.030	0.502	0.269–0.937
Expansion rate after initial ballooning (%)	−0.028	0.011	−2.552	0.015	0.972	0.950–0.995
Calcium antagonists	−0.800	0.345	−2.380	0.020	0.449	0.228–0.884

### Nomogram prediction model

In this study, we combined the aforementioned risk factors to develop an integrated predictive model for vascular restenosis following PCI in patients with CAD and DM.

The model exhibited good discrimination, achieving an apparent AUC of 0.833 (Fig. 1). Internal validation by bootstrap resampling (1,000 replications) yielded an optimism-corrected AUC of 0.808, indicating that the model performance was not substantially overestimated.

**Figure 1:**
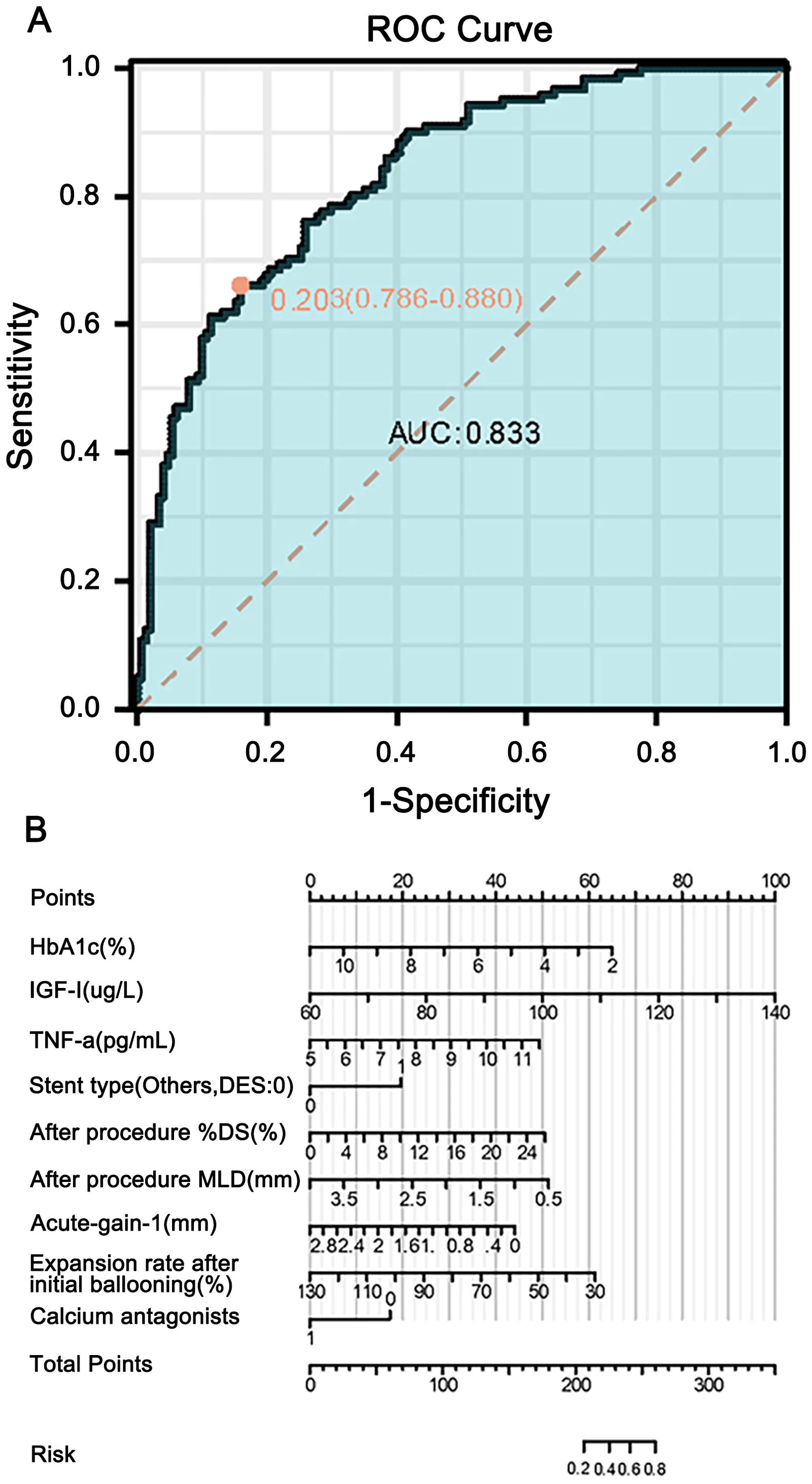
Nomogram prediction model. (A) ROC curve; (B) nomogram.

## Discussion

In this study, we presented a comprehensive analysis of serum metabolites and clinical risk factors to develop a nomogram prediction model for post-PCI vascular restenosis in CAD patients with DM. One of the key findings from our study was the role of metabolic factors, particularly those associated with glycemic control and lipid metabolism, in influencing restenosis risk. Lower glycated hemoglobin (HbA1c) levels were inversely linked to restenosis, suggesting that suboptimal glucose metabolism may not be the primary driver of neointimal hyperplasia in this cohort (Dar et al., 2020; Yang et al., 2020). This counterintuitive association should, however, be interpreted with considerable caution. Because all participants had established T2DM and were managed with glucose-lowering therapy, a substantial proportion in both groups had attained HbA1c values below the 6.5% diagnostic threshold at the time of measurement, reflecting treatment status rather than a reclassification of diabetes status. More importantly, as this was a retrospective observational study, the inverse HbA1c-restenosis association is susceptible to residual confounding and possible reverse causation: patients with more advanced or aggressive coronary disease may have received more intensive risk-factor management, including tighter glycemic control, which could spuriously link lower HbA1c with adverse outcomes. We were unable to fully adjust for diabetes duration, the specific class and intensity of hypoglycemic medication, or time-in-range, and these unmeasured factors may contribute to the observed relationship. This finding should therefore be regarded as hypothesis-generating and requires confirmation in prospective studies with detailed glycemic phenotyping. While one might initially expect poorer glycemic control to exacerbate restenosis due to enhanced lipid oxidation and inflammatory responses, our findings align with a growing body of literature suggesting that rigorous glycemic control alone might not suffice to mitigate restenosis risk (Pehrson et al., 2023; Otto et al., 2022). This underscores the complex interrelation where diabetic vasculopathy may not solely hinge on glucose levels, but also on other factors, such as insulin resistance and dyslipidemia (Muramatsu et al., 2023).

Lipid metabolism also showed significant association with restenosis, exemplified by increased levels of LDL cholesterol in the restenosis group. Elevated LDL levels potentiate endothelial dysfunction and inflammatory processes, which were central to the pathophysiology of atherosclerosis and by extension, restenosis (Mahmoud et al., 2024; Lugo-Gavidia, Alcocer-Gamba & Martinez-Cervantes, 2024). The alteration of lipid profiles in diabetes further accentuates lipid-driven vascular injury through oxidative stress and endothelial cell activation, highlighting the need for aggressive lipid management in this population (Liu et al., 2023). Inflammatory biomarkers, such as TNF-α and high-sensitivity C-reactive protein (hsCRP), also emerged as significant predictors of restenosis. These markers indicate a persistent inflammatory state in patients who develop restenosis, reflecting a milieu that promotes neointimal proliferation and maladaptive vascular remodeling (Lin et al., 2022; Liang et al., 2023). TNF-α, a potent mediator of inflammation, can induce smooth muscle cell proliferation and migration, hallmarks of restenotic lesions (Levent et al., 2022). The link between systemic inflammation and localized vascular responses highlights the systemic nature of CAD and its implications in procedural outcomes like restenosis (Lai et al., 2021). Elevated levels of TNF-α and hsCRP are linked to a greater risk of vascular restenosis following PCI in patients with CAD and DM. Studies have shown that elevated levels of IGF-I are closely associated with the occurrence of in-stent restenosis following PCI. IGF-I plays a crucial role in cell growth and proliferation, and increased expression may indicate enhanced activity of vascular smooth muscle cells, thereby promoting excessive neointimal hyperplasia. By focusing on the role of IGF-I, researchers can gain deeper insights into how it influences the vascular healing process and explore effective means to inhibit inappropriate cellular proliferation, thus reducing the risk of restenosis after PCI in patients with CAD and DM (Chikata et al., 2022; Song et al., 2022).

Our procedural findings align with established knowledge that specific PCI-related factors, such as stent type and residual stenosis post-PCI, significantly impact restenosis rates. The use of DES was more prevalent in the no-restenosis group, was consistent with the previous report that DES was benefit in reducing restenosis risk (Greco et al., 2025). DES reduces neointimal hyperplasia through localized drug delivery, and thus decreasing the risk of restenosis. However, some studied indicated that factors like drug resistance or variability in patients may limit the benefit of DES (Jeger et al., 2020; Ito et al., 2022). Therefore, the use of DES may need to be individualized. A postoperative assessment determined that greater residual stenosis and reduced acute lumen gain are strongly linked to a higher likelihood of vascular restenosis following PCI in CAD patients with diabetes (Doenst et al., 2024). These factors not only reflect suboptimal outcomes during the procedure but may also be attributed to inherent patient pathologic characteristics, such as plaque properties and vessel compliance, which can affect long-term patency of the stented segment. Specifically, higher residual stenosis indicates that the procedure failed to adequately dilate the lesion site, potentially leading to altered local hemodynamics, promoting excessive neointimal hyperplasia, and thereby increasing the risk of restenosis (Shah & Maiolo, 2021; Sharma et al., 2024). Lower acute lumen gain suggests that the procedure did not effectively restore sufficient lumen area, subjecting the vessel wall to greater stress, thus stimulating smooth muscle cell migration and proliferation, further promoting the occurrence of restenosis.

Interestingly, studies have found a lower usage rate of calcium channel blockers (CCBs) in the restenosis group, suggesting that CCBs may have a potential protective role in preventing vascular restenosis following PCI (Lauder et al., 2023; Matta et al., 2020). Mechanistically, calcium channel blockers can regulate vascular tone by inhibiting calcium ion influx into smooth muscle cells and may attenuate calcium-mediated proliferative signaling, processes that have been proposed to influence neointimal formation; these mechanisms offer a biologically plausible basis for the observed association. Nevertheless, this association must be interpreted with caution. The present study was a retrospective, case-control analysis in which calcium channel blockers were not randomly allocated, and we did not adjust for the indications for their prescription. Patients receiving calcium channel blockers may have differed systematically from non-users in baseline characteristics such as blood pressure, severity of coronary disease, or concurrent cardioprotective medication, any of which could independently affect restenosis risk. Confounding by indication therefore cannot be excluded, and our observational data cannot establish that calcium channel blockers causally prevent restenosis or reduce neointimal hyperplasia. Accordingly, the protective association we report should be regarded as hypothesis-generating, and dedicated studies incorporating propensity-score adjustment or randomized designs are needed to clarify the potential role of calcium channel blockers in post-PCI management, particularly in patients with CAD and DM (Sawano et al., 2020; Schindler et al., 2023).

Through correlation analysis and logistic regression analysis, we have defined HbA1c, IGF-I, LDL cholesterol, TNF-α, hsCRP, stenting total length, stent type, post-procedure %DS, post-procedure MLD, acute gain-1, expansion rate after initial ballooning, and the use of calcium antagonists as influencing factors for post-PCI vascular restenosis in patients with CAD and DM. Based on these factors, we constructed a nomogram to predict post-PCI vascular restenosis in this patient population. The nomogram was constructed using the predictors that retained statistical significance in the multivariable model, and yielded an apparent AUC of 0.833. To address the modest events-per-variable ratio (approximately 10) and the optimism inherent in evaluating a model on the same dataset used for its development, we performed internal validation using bootstrap resampling (1000 replications). After optimism correction, the AUC was 0.808, indicating that the discriminative ability remained acceptable and was not substantially inflated. For context, previously reported models by Smith et al. (AUC = 0.752), Jones et al. (AUC = 0.785), and Lee et al. (AUC = 0.801) reported AUC values in a broadly comparable range (Capodanno et al., 2023; Liang, He & Gu, 2021; Moura et al., 2023). However, because these AUC values were derived from different populations and outcome definitions and were not obtained under a common validation framework, this comparison should be interpreted with caution and does not by itself establish the superiority of our model. Rather, the bootstrap-corrected performance suggests that our nomogram offers reasonable discrimination that is consistent with previously reported models. This indicates that, pending external validation, our nomogram may serve as a useful exploratory tool to support risk stratification and management of post-PCI vascular restenosis in this population (Persson et al., 2023). Our study offers valuable insights into the multifaceted predictors of post-PCI vascular restenosis; however, several limitations must be acknowledged. One primary concern is the retrospective design, which may introduce selection bias and constrain the ability to assess causal relationships. The study cohort, though adequately sized, was derived from a single center, potentially affecting the generalizability of our findings to broader and more diverse populations. Additionally, our reliance on serum metabolites and clinical data confines the scope of identified predictors, potentially overlooking other genetic or environmental factors influencing restenosis. From a modeling perspective, the number of restenosis events relative to the number of candidate predictors yielded an events-per-variable ratio of approximately 10, which is at the lower limit generally regarded as acceptable and increases the risk of model optimism and overfitting. Although we performed bootstrap internal validation and reported an optimism-corrected AUC, we did not apply penalized variable-selection methods such as LASSO, and no external or split-sample validation was undertaken; the reported discrimination may therefore still be somewhat optimistic. In addition, our dataset did not reliably distinguish emergency from elective PCI, so we were unable to perform a stratified analysis by procedural timing. Given that inflammatory biomarker profiles can differ between emergency and elective procedures, this represents a potential source of unmeasured confounding that should be addressed in future work. The associations observed for individual predictors, including the inverse association of HbA1c and the apparent protective association of calcium antagonists, may also be affected by residual confounding and should be interpreted as hypothesis-generating. Lastly, the nomogram, although showing acceptable discrimination on internal validation, requires prospective validation in independent cohorts to confirm its clinical applicability and reliability across varied settings. Future studies should aim to address these limitations through a multicenter approach and incorporate a broader spectrum of data to refine the predictive model further.

## Conclusion

In conclusion, our study has comprehensively explored the determinants of restenosis following percutaneous coronary intervention (PCI), revealing the complex interplay of various factors influencing outcomes in CAD patients with diabetes. We identified serum metabolites and biomarkers (including HbA1c, IGF-I, LDL cholesterol, TNF-α, and hsCRP) as well as procedural factors (stenting total length, stent type, post-procedure %DS, post-procedure MLD, acute gain-1, expansion rate after initial ballooning), and the use of calcium antagonists as predictors of restenosis after PCI. The nomogram showed good discrimination on internal bootstrap validation; however, as this was a single-center retrospective analysis without external validation, the model should be regarded as exploratory and hypothesis-generating rather than as a ready-to-use clinical decision tool. Prospective, multicenter external validation is required before clinical application. As our study elucidates, the multifactorial nature of restenosis underscores the need for an integrated approach that combines pharmacological treatment and procedural optimization. Tailored therapies targeting metabolic and inflammatory pathways, along with meticulous surgical planning and execution, are crucial for reducing the risk of restenosis. Future research may focus on, exploring new anti-inflammatory or metabolic therapies, and improving intervention techniques to enhance procedural success and durability.

## Supplemental Information

10.7717/peerj.21697/supp-1 Supplemental Information 1 Data and codebook

10.7717/peerj.21697/supp-2 Supplemental Information 2 STROBE checklist

## Abbreviations

ACE: angiotensin-converting enzyme
ARB: angiotensin receptor blocker
AUC: area under the curve
BMI: body mass index
CAD: coronary artery disease
CI: confidence interval
DES: drug-eluting stent
DM: diabetes mellitus
HbA1c: glycated hemoglobin
HDL-C: high-density lipoprotein cholesterol
hsCRP: high-sensitivity C-reactive protein
IGF-I: insulin-like growth factor I
LDL-C: low-density lipoprotein cholesterol
MLD: minimal lumen diameter
OR: odds ratio
PCI: percutaneous coronary intervention
RD: reference diameter
ROC: receiver operating characteristic
SD: standard deviation
SPSS: Statistical Package for the Social Sciences
T2DM: type 2 diabetes mellitus
TG: triglycerides
TNF-α: tumor necrosis factor alpha
VSMC: vascular smooth muscle cell
%DS: percentage diameter stenosis

## Additional Information and Declarations

### Data Availability

The following information was supplied regarding data availability: The raw measurements are available in the Supplemental Files.
