# Supplementary material for: Nomogram for predicting post-percutaneous coronary intervention restenosis in patients with coronary artery disease and diabetes: a retrospective study
Source: PeerJ. 2026 Oct 13;14:e21697. doi: 10.7717/peerj.21697 (PMC13674360; doi:10.7717/peerj.21697)
Supplement: Supplementary file 1 [file peerj-14-21697-s002.doc]

STROBE Statement—checklist of items that should be included in reports of observational studies

|  | Item No | Recommendation | Relevant text from manuscript |
| --- | --- | --- | --- |
| **Title and abstract** | 1 | (*a*) Indicate the study’s design with a commonly used term in the title or the abstract | Page 1: line 1-3.  Page 2: 23-29 |
|  |  | (*b*) Provide in the abstract an informative and balanced summary of what was done and what was found | Page 1: line 17-25; Page 2: line 26-45 |
| Introduction | | |  |
| Background/rationale | 2 | Explain the scientific background and rationale for the investigation being reported | Page 3: line 51-63; Page 4: line 76-112 |
| Objectives | 3 | State specific objectives, including any prespecified hypotheses | Page 5: line 107-112 |
| Methods | | |  |
| Study design | 4 | Present key elements of study design early in the paper | Page 5: line 107-108; line 115-116 |
| Setting | 5 | Describe the setting, locations, and relevant dates, including periods of recruitment, exposure, follow-up, and data collection | Page 5: line 116-120 |
| Participants | 6 | (*a*) *Cohort study*—Give the eligibility criteria, and the sources and methods of selection of participants. Describe methods of follow-up  *Case-control study*—Give the eligibility criteria, and the sources and methods of case ascertainment and control selection. Give the rationale for the choice of cases and controls  *Cross-sectional study*—Give the eligibility criteria, and the sources and methods of selection of participants | *Page 5: line 121-125; Page 6: line 126-142* |
|  |  | (*b*) *Cohort study*—For matched studies, give matching criteria and number of exposed and unexposed  *Case-control study*—For matched studies, give matching criteria and the number of controls per case | *N/A (unmatched case-control study)* |
| Variables | 7 | Clearly define all outcomes, exposures, predictors, potential confounders, and effect modifiers. Give diagnostic criteria, if applicable | Page 6: line 137-142; Page 5: line 123-124 |
| Data sources/ measurement | 8* | For each variable of interest, give sources of data and details of methods of assessment (measurement). Describe comparability of assessment methods if there is more than one group | *Page 8: line 182-199; Page 9: line 200-212* |
| Bias | 9 | Describe any efforts to address potential sources of bias | Page 5: line 116-125; Page 6: line 128-136 |
| Study size | 10 | Explain how the study size was arrived at | Page 5: line 116; Page 6: line 141-142 |
| Quantitative variables | 11 | Explain how quantitative variables were handled in the analyses. If applicable, describe which groupings were chosen and why | Page 10: line 230-246 |
| Statistical methods | 12 | (*a*) Describe all statistical methods, including those used to control for confounding | Page 10: line 230-246 |
|  |  | (*b*) Describe any methods used to examine subgroups and interactions | N/A (not applicable) |
|  |  | (*c*) Explain how missing data were addressed | N/A (not reported) |
|  |  | (*d*) *Cohort study*—If applicable, explain how loss to follow-up was addressed  *Case-control study*—If applicable, explain how matching of cases and controls was addressed  *Cross-sectional study*—If applicable, describe analytical methods taking account of sampling strategy | *N/A (not applicable)* |
|  |  | (*e*) Describe any sensitivity analyses | N/A (not applicable) |

Continued on next page

| Results | | |  |
| --- | --- | --- | --- |
| Participants | 13* | (a) Report numbers of individuals at each stage of study—eg numbers potentially eligible, examined for eligibility, confirmed eligible, included in the study, completing follow-up, and analysed | Page 5: line 116; Page 6: line 141-142; Page 11: line 250-251 |
|  |  | (b) Give reasons for non-participation at each stage | N/A (not reported) |
|  |  | (c) Consider use of a flow diagram | N/A (no flow diagram provided) |
| Descriptive data | 14* | (a) Give characteristics of study participants (eg demographic, clinical, social) and information on exposures and potential confounders | Page 11: line 250-269 |
|  |  | (b) Indicate number of participants with missing data for each variable of interest | N/A (not reported) |
|  |  | (c) *Cohort study*—Summarise follow-up time (eg, average and total amount) | N/A (not applicable, case-control study) |
| Outcome data | 15* | *Cohort study*—Report numbers of outcome events or summary measures over time | *N/A (not applicable, case-control study)* |
|  |  | *Case-control study—*Report numbers in each exposure category, or summary measures of exposure | *Page 11: line 250-251; Page 12: line 271-285; Page 13: line 288-299* |
|  |  | *Cross-sectional study—*Report numbers of outcome events or summary measures | *N/A (not applicable)* |
| Main results | 16 | (*a*) Give unadjusted estimates and, if applicable, confounder-adjusted estimates and their precision (eg, 95% confidence interval). Make clear which confounders were adjusted for and why they were included | Page 15: line 346-367; Page 16: line 369-387; Page 17: line 388-415 |
|  |  | (*b*) Report category boundaries when continuous variables were categorized | N/A (continuous variables not categorized) |
|  |  | (*c*) If relevant, consider translating estimates of relative risk into absolute risk for a meaningful time period | N/A (not applicable) |
| Other analyses | 17 | Report other analyses done—eg analyses of subgroups and interactions, and sensitivity analyses | Page 15: line 346-367; Page 16: line 369-387 |
| Discussion | | |  |
| Key results | 18 | Summarise key results with reference to study objectives | Page 18: line 421-434; Page 21: line 498-511 |
| Limitations | 19 | Discuss limitations of the study, taking into account sources of potential bias or imprecision. Discuss both direction and magnitude of any potential bias | Page 21: line 511-518; Page 22: line 519-523 |
| Interpretation | 20 | Give a cautious overall interpretation of results considering objectives, limitations, multiplicity of analyses, results from similar studies, and other relevant evidence | Page 18: line 421-443; Page 21: line 498-523 |
| Generalisability | 21 | Discuss the generalisability (external validity) of the study results | Page 21: line 514-518; Page 22: line 519-523 |
| Other information | | |  |
| Funding | 22 | Give the source of funding and the role of the funders for the present study and, if applicable, for the original study on which the present article is based | Page 24: line 588-589 |

*Give information separately for cases and controls in case-control studies and, if applicable, for exposed and unexposed groups in cohort and cross-sectional studies.

**Note:** An Explanation and Elaboration article discusses each checklist item and gives methodological background and published examples of transparent reporting. The STROBE checklist is best used in conjunction with this article (freely available on the Web sites of PLoS Medicine at http://www.plosmedicine.org/, Annals of Internal Medicine at http://www.annals.org/, and Epidemiology at http://www.epidem.com/). Information on the STROBE Initiative is available at www.strobe-statement.org.
